# Supplementary material for: Myocarditis in Athletes Recovering from COVID-19: A Systematic Review and Meta-Analysis
Source: Int J Environ Res Public Health. 2022 Apr 2;19(7):4279. doi: 10.3390/ijerph19074279 (PMC8998516; doi:10.3390/ijerph19074279)
Supplement: Supplementary file 1 [file ijerph-19-04279-s001.zip › ijerph-1622987-supplementary.pdf]

# Tables and Figures

Table S1. Research strategy.

| Database Searched | N.° | Search Words | Search Strategy | Results |
|---|---|---|---|---|
| PubMed | 1 | COVID-19 Myocarditis athletes | ("COVID-19"[All Fields] OR "COVID-2019"[All Fields] OR "severe acute respiratory syndrome coronavirus 2"[Supplementary Concept] OR "severe acute respiratory syndrome coronavirus 2"[All Fields] OR "2019-nCoV"[All Fields] OR "SARS-CoV-2"[All Fields] OR "2019nCoV"[All Fields] OR (("Wuhan"[All Fields] AND ("coronavirus"[MeSH Terms] OR "coronavirus"[All Fields])) AND (2019/12[PDAT] OR 2020[PDAT]))) AND ("myocarditis"[MeSH Terms] OR "athletes"[All Fields]) | 52 |
| PubMed | 2 | Sars-CoV-2 Myocarditis athletes | ("COVID-19"[All Fields] OR "COVID-2019"[All Fields] OR "severe acute respiratory syndrome coronavirus 2"[Supplementary Concept] OR "severe acute respiratory syndrome coronavirus 2"[All Fields] OR "2019-nCoV"[All Fields] OR "SARS-CoV-2"[All Fields] OR "2019nCoV"[All Fields] OR (("Wuhan"[All Fields] AND ("coronavirus"[MeSH Terms] OR "coronavirus"[All Fields])) AND (2019/12[PDAT] OR 2020[PDAT]))) AND ("myocarditis"[MeSH Terms] OR "athletes"[All Fields]) | 38 |
| PubMed | 3 | COVID-19 CMR athletes | ("COVID-19"[All Fields] OR "COVID-2019"[All Fields] OR "severe acute respiratory syndrome coronavirus 2"[Supplementary Concept] OR "severe acute respiratory syndrome coronavirus 2"[All Fields] OR "2019-nCoV"[All Fields] OR "SARS-CoV-2"[All Fields] OR "2019nCoV"[All Fields] OR (("Wuhan"[All Fields] AND ("coronavirus"[MeSH Terms] OR "coronavirus"[All Fields])) AND (2019/12[PDAT] OR 2020[PDAT]))) AND ("CMR[MeSH Terms] OR "CMR"[All Fields] OR "athletes"[All Fields]) | 17 |
| PubMed | 4 | Sars-CoV-2 CMR athletes | ("COVID-19"[All Fields] OR "COVID-2019"[All Fields] OR "severe acute respiratory syndrome coronavirus 2"[Supplementary Concept] OR "severe acute respiratory syndrome coronavirus 2"[All Fields] OR "2019-nCoV"[All Fields] OR "SARS-CoV-2"[All Fields] OR "2019nCoV"[All Fields] OR (("Wuhan"[All Fields] AND ("coronavirus"[MeSH Terms] OR "coronavirus"[All Fields])) AND (2019/12[PDAT] OR 2020[PDAT]))) AND ("CMR"[MeSH Terms] OR "CMR"[All Fields] OR "athletes"[All Fields]) | 14 |
| PubMed | 5 | COVID-19 Pericardial effusion athletes | ("COVID-19"[All Fields] OR "COVID-2019"[All Fields] OR "severe acute respiratory syndrome coronavirus 2"[Supplementary Concept] OR "severe acute respiratory syndrome coronavirus 2"[All Fields] OR "2019-nCoV"[All Fields] OR "SARS-CoV-2"[All Fields] OR "2019nCoV"[All Fields] OR (("Wuhan"[All Fields] AND ("coronavirus"[MeSH Terms] OR "coronavirus"[All Fields])) AND (2019/12[PDAT] OR 2020[PDAT]))) AND ("pericardial effusion"[MeSH Terms] OR ("athletes"[All Fields] AND "athletes[All Fields]) OR "pericardial"[All Fields] OR "athletes"[All Fields]) | 4 |
| PubMed | 6 | Sars-CoV-2 Pericardial effusion athletes | ("COVID-19"[All Fields] OR "COVID-2019"[All Fields] OR "severe acute respiratory syndrome coronavirus 2"[Supplementary Concept] OR "severe acute respiratory syndrome coronavirus 2"[All Fields] OR "2019-nCoV"[All Fields] OR "SARS-CoV-2"[All Fields] OR "2019nCoV"[All Fields] OR (("Wuhan"[All Fields] AND ("coronavirus"[MeSH Terms] OR "coronavirus"[All Fields])) AND (2019/12[PDAT] OR 2020[PDAT]))) AND ("pericardial effusion"[MeSH Terms] OR ("athletes"[All Fields] AND "athletes"[All Fields]) OR "pericardial"[All Fields] OR "athletes"[All Fields]) | 4 |
| PubMed | 6 | | Results | 129 |
| SCOPUS | 1 | COVID-19 Myocarditis athletes | (Covid 19 myocarditis AND athletes) | 54 |
| SCOPUS | 2 | Sars-CoV-2 Myocarditis athletes | (Sars-Cov-2 AND myocarditis AND athletes) | 24 |
| SCOPUS | 2 | | Results | 78 |
| Web of Science | 1 | COVID-19 Myocarditis athletes | (Covid 19 myocarditis athletes) | 35 |
| Web of Science | 2 | Sars-CoV-2 Myocarditis athletes | (Sars-Cov-2 myocarditis athletes) | 11 |
| Web of Science | 2 | | Results | 46 |
| Total | | | Results | 253 |

Table S2. Critical appraisal included cohort studies

| | Clark et al. (2021) | Cavigli et al. (2021) | Cavigli et al. (2021) | Daniels et al. (2021) | Hendrickson et al. (2021) | Malek et al. (2021) | Moulson et al. (2021) | Guevarra et al. (2022) | Vago et al. (2020) | Mascia et al. (2020) |
|---|---|---|---|---|---|---|---|---|---|---|
| 1 Similar groups | + | - | - | - | - | - | - | - | + | + |
| 2 Exposures similar in different groups | - | - | - | - | - | - | - | - | - | - |
| 3 Exposures validly measured | + | - | - | + | + | + | + | + | + | - |
| 4 Identification confounding factors | - | - | - | - | - | - | - | - | - | - |
| 5 Strategies confounding factors | - | - | - | - | - | - | - | - | - | - |
| 6 Participants free of outcome at the start | - | - | - | - | - | - | - | - | - | - |
| 7 Outcomes validly measured | - | + | + | + | + | + | + | + | + | + |
| 8 Follow-up long enough | - | - | - | + | - | - | + | - | - | - |
| 9 Follow-up complete or reasons described | - | - | - | - | - | - | + | - | - | - |
| 10 Strategies incomplete follow-up utilized | - | - | - | - | - | - | - | - | - | - |
| 11 Appropriate statistical analysis | + | + | + | + | + | + | + | + | + | + |
| Total yes answers: | 3 | 2 | 2 | 4 | 3 | 3 | 5 | 3 | 4 | 3 |
| ('+' = yes, '-'= no, and ' '= not applicable) according to the Joanna Briggs Institute Critical Appraisal tools checklists. | | | | | | | | | | |

Table S3. Critical appraisal included Case control studies

| | Gervasi et al. (2020) |
|---|---|
| 1 Comparable group | + |
| 2 Cases and controls matched | + |
| 3 Same criteria used for identification | + |
| 4 Exposure measured in a standard, valid way | + |
| 5 Exposure measured in the same way for both groups | + |
| 6 Confounding factors | - |
| 7 Strategies deal with confounding factors | - |
| 8 Outcomes validly measured | + |
| 9 Exposure period long enough | + |
| 10 Appropriate statistical analysis Total yes answers: | 8 |
| ('+' = yes, '-'= no, and ' '= not applicable) according to the Joanna Briggs Institute Critical Appraisal tools checklists. | |

Table S4. Critical appraisal included Cross sectional studies

| | Brito et al. (2020) | Martinez et al. (2021) | Liliána Szabó et al. (2021) |
|---|---|---|---|
| 1 Inclusion criteria clearly defined | - | - | - |
| 2 Study subjects and setting described in detail | - | - | + |
| 3 Exposure measured in a standard, valid way | + | + | + |
| 4 Objective, standard criteria used for measurement of the condition? | - | + | + |
| 5 Confounding factors | - | - | - |
| 6 Strategies deal with confounding factors | - | - | - |
| 7 Outcomes validly measured | - | + | + |
| 8 Appropriate statistical analysis | + | - | + |
| Total yes answers: | 2 | 3 | 5 |
| ('+' = yes, '-'= no, and ' '= not applicable) according to the Joanna Briggs Institute Critical Appraisal tools checklists. | | | |

Table S5. Critical appraisal included case series

| | Starekova et al. (2021) |
|---|---|
| 1 Inclusion criteria clearly defined | - |
| 2 Condition measured in a standard, valid way | + |
| 3 Valid methods used for identification of the condition | - |
| 4 Consecutive inclusion | + |
| 5 Complete inclusion | + |
| 6 Clear reporting of demographics | + |
| 7 Clear reporting of clinical information | - |
| 8 Outcomes clearly reported | + |
| 9 Clear reporting of presenting clinic demographic information | - |
| 10 Appropriate statistical analysis | + |
| Total yes answers: | 6 |
| ('+' = yes, '-'= no, and ' '= not applicable) according to the Joanna Briggs Institute Critical Appraisal tools checklists. | |

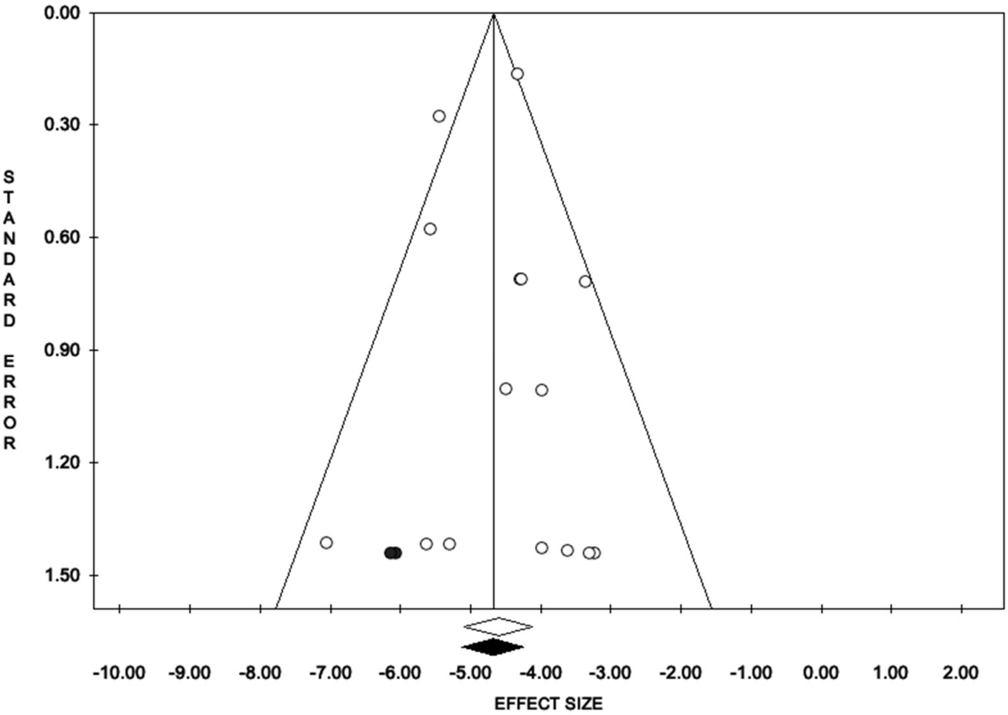


**Figure S1:** Funnel plot showing publication bias on myocarditis in total sample of athletes (n° 7988) who recovered from COVID-19 infection. Egger's linear regress test was not significant (p-value 0.98)

Tables and Figures legend

TAB. s1 Research strategy

TAB. s2-5 Full Quality Assesetement JBI

FIG. s1 Funnel plot showing publication bias on myocarditis in total sample of athletes (n° 7988) who recovered from COVID-19 infection. Egger's linear regress test was not significant (p-value 0.98)
